# A qualitative study of barriers to care-seeking for diabetic foot ulceration across multiple levels of the healthcare system

**DOI:** 10.1186/s13047-022-00561-4

**Published:** 2022-08-06

**Authors:** Tze-Woei Tan, Rebecca M. Crocker, Kelly N. B. Palmer, Chris Gomez, David G. Armstrong, David G. Marrero

**Affiliations:** 1 Southwestern Academic Limb Salvage Alliance (SALSA), Los Angeles Tucson, USA; 2 Division of Vascular Surgery and Endovascular Therapy, Keck School of Medicine, University of Southern California, 1520 San Pablo Street, Ste 4300, Los Angeles, CA 90033 USA; 3 Center for Health Disparities Research (CHDR), University of Arizona Health Sciences, Tucson, AZ USA; 4 University of Arizona College of Medicine, Tucson, AZ USA

**Keywords:** Diabetic foot complications, Foot ulceration, Barriers in assessing medical care, Health care system barriers, Qualitative

## Abstract

**Introduction:**

The mechanisms for the observed disparities in diabetes-related amputation are poorly understood and could be related to access for diabetic foot ulceration (DFU) care. This qualitative study aimed to understand patients’ personal experiences navigating the healthcare system and the barriers they faced.

**Methods:**

Fifteen semi-structured interviews were conducted over the phone between June 2020 to February 2021. Participants with DFUs were recruited from a tertiary referral center in Southern Arizona. The interviews were audio-recorded and analyzed according to the NIMHD Research Framework, focusing on the health care system domain.

**Results:**

Among the 15 participants included in the study, the mean age was 52.4 years (66.7% male), 66.7% was from minority racial groups, and 73.3% was Medicaid or Indian Health Service beneficiaries. Participants frequently reported barriers at various levels of the healthcare system.

On the individual level, themes that arose included health literacy and inadequate insurance coverage resulting in financial strain. On the interpersonal level, participants complained of fragmented relationships with providers and experienced challenges in making follow-up appointments. On the community level, participants reported struggles with medical equipment.

On the societal level, participants also noted insufficient preventative foot care and education before DFU onset, and many respondents experienced initial misdiagnoses and delays in receiving care.

**Conclusions:**

Patients with DFUs face significant barriers in accessing medical care at many levels in the healthcare system and beyond. These data highlight opportunities to address the effects of diabetic foot complications and the inequitable burden of inadequately managed diabetic foot care.

## Introduction

Diabetic foot ulceration (DFU) is a common and often catastrophic complication for people with diabetes. In the United States, people with diabetes have an up to 34% lifetime risk of developing a foot ulcer [1, 2], a medical complication that increases their five-year mortality rate by 2.5 times [3, 4]. Moreover, foot ulceration is a causal factor for up to 85% of diabetic patients who subsequently undergo lower extremity amputation [1, 5]. As compared to the overall United States population, people with diabetes are more likely to undergo lower extremity amputation and repeat amputations [1, 6]. The annual medical cost associated with DFU care in the United States is an additional $9–13 billion on top of other costs associated with diabetes [7].

Moreover, DFUs and subsequent amputations are unevenly patterned along lines of racial and ethnic minority status, low socio-economic status, low insurance coverage rates, and geographic isolation. African American, Hispanic, and Native American adults with diabetes have higher prevalence of DFUs and amputation than their White counterparts [8–10]. Across the board, patients in the lowest income quartiles experience higher odds of amputation and death due to peripheral artery disease [11, 12]. In addition, those with suboptimal or no medical insurance are at an elevated risk of major amputation [13]. This illuminates a glaring and yet unabated public health problem, especially among minority and low-income populations [8, 9, 12–16].

The mechanisms of these observed disparities in DFU incidence and progression are poorly understood [9, 11, 17, 18]. There is evidence, however, indicating that access to affordable and quality medical care, preventive services, and limb salvage care is an important contributing factor to disparities in amputation rates [19–21]. This qualitative study aimed to understand patients’ personal experiences with DFUs in a safety net health system, including their processes of navigating the healthcare system and the barriers they faced. The themes elicited in the study concerning multiple barriers at varying levels of the healthcare system will help to improve health care delivery in a population experiencing elevated risks of diabetes-related ulceration and amputation.

## Methods

### Design

This qualitative study was designed to better understand the various challenges faced by patients with a history of DFUs and lower extremity amputations as they managed their conditions and sought medical care. Semi-structured interviews were conducted between June 2020 to February 2021 and the results were analyzed according to the “Health Care System” domain of the National Institute on Minority Health and Health Disparities Research Framework [22]. The University of Arizona Institutional Review Board approved the study in July 2019 (Protocol Number 1906749805).

### Participants

Patients were selected from the Southwestern Academic Limb Salvage Alliance (SALSA), a multidisciplinary limb salvage care team located in Tucson, Arizona, to participate in semi-structured interviews. SALSA treats over 5,000 patient visits annually for diabetic foot problems, of which 40% are from racial and ethnic minority groups. It is the primary referral center for limb salvage and care for minorities and patients with low socioeconomic status in suburban and rural Arizona. Participants were identified and approached for participation during scheduled clinic appointments or by follow-up phone calls by our research team. We purposely sampled participants, using criterion sampling, to reflect the diverse range of race/ethnicity, gender, history of DFU, foot infection, minor amputation (below the ankle), and major amputation (ankle or above) treated by SALSA [23].

### Interview guide and data collection

The research team jointly developed a semi-structured interview guide to encourage patient perspectives regarding their living experiences with foot ulceration and how they sought care for DFUs. Interviews were conducted in the patients’ preferred language (English or Spanish). Three research team members experienced in qualitative interviews (R.M.C., K.N.B.P., and D.G.M.) completed 15 interviews over the phone, lasting 40–60 min each. Interviews were recorded with consent using the “Tape A Call” mobile application (www.tapeacall.com) or via the University of Arizona Health Sciences Zoom Platform. The interviews were conducted in phases to allow for simultaneous analysis and redirection of subsequent data collection.

### Analysis

The research team used the Dedoose software version 9.0.17 (SocioCultural Research Consultants, LLC, Los Angeles, CA) to assist in data storage, coding, and data analysis. Audio files of the interviews were transcribed into the language spoken. After a quality assurance check, the transcriptions were uploaded into the software. The transcripts were independently reviewed and coded by three members of the research team (R.M.C., K.N.B.P., and T-W.T.). Data for this article were analyzed according to the NIMHD Research Framework (2017) that includes a multilevel approach including individual, interpersonal, community, and societal-level factors. While this model includes several domains, for the purposes of this paper we are focusing only on the Health Care System domain. This framework has been used in health disparities research to conceptualize and evaluate a wide array of determinants that promote or worsen health disparities [24]. Team members met regularly to compare coding results and resolve discrepancies by discussion and consensus.

## Results

**Fig. 1:**
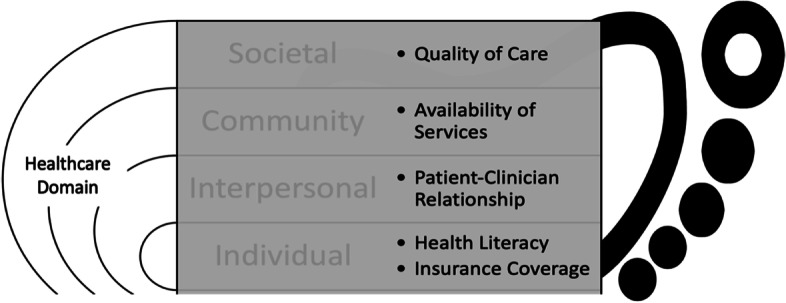
Patient reported barriers at all levels influence of the health care system domain

The study sample included 15 participants (Table 1). The mean age was 54.2 years. Eleven participants (73.3%) were Medicaid or Indian Health Program beneficiaries and 80% of participants were either unemployed or had retired. All participants had history of at least one DFU, 12 had a history of foot infection, eight underwent minor amputations, and one had a major amputation. Four patients underwent at least one open surgery or endovascular procedure due to peripheral artery disease. During the interviews, participants frequently reported barriers at various levels of the health care system (Table 2, Fig. 1).

**Table 1:** Baseline demographics and comorbidities of the participants

	*N* = 15
Age, year	54.2
Gender, n (%)	
Male	10 (66.7%)
Female	5 (33.3%
Race and ethnicity, n (%)	
White	5 (33.3%)
Native American	5 (33.3%)
Hispanic	5 (33.3%)
Primary Insurance	
Commercial	1 (6.7%)
Medicare	3 (20.0%)
Medicaid of Indian Health	11 (73.3%)
Employment Status	
Employed	3 (20.0%)
Unemployed	7 (46.7%)
Retired	5 (33.3%)
History of Diabetic Foot Ulceration	15 (100.0%)
History of Diabetic Foot Infection	12 (80.0%)
History of Peripheral Artery Disease	7 (46.7%)
Open surgery or endovascular procedure	4 (26.7%)
History of Minor Amputation	8 (53.3%)
History of Major Amputation	1 (6.7%)

**Table 2:** Quotes from the interviews categorized according to the National Institute on Minority Health and Health Disparities Research Framework

**Individual Level of Influence**	
Health Literacy	“Nobody ever really said what I’m looking for just anything that is not normal, I guess. But like I said, I have never heard of a diabetic foot ulcer.” (57-year-old Hispanic male, history of DFU)
	If I had gotten better, like a different type of information that they could’ve given me, that might’ve helped me improve this ulcer to be going away. From what I have been given, you know, it’s just hard. I don’t know if it’s my foot itself or if it’s the medication. I don’t know. I don’t know if I am a unique case, I know there are people out there that have one foot. And they are able to get, probably, their ulcer better” (29-year-old Native female, history of DFU and recurrent foot infection)
Insurance Coverage	“I was in the hospital for 15 days, 13 days. They are charging me a copay, but I don’t have money to pay it. I am currently not working. I have social security and they don’t give me very much and it’s not enough to cover the copay.” (67-year-old Hispanic male, commercial insurance)
	“They want me to get diabetic shoes and the orthotic but at the time I didn’t have Medicaid … and with the deductible, they wanted $1,000 for the pair of shoes and the orthotic and I couldn’t afford it.” (45-year-old White female, Medicaid)
**Interpersonal Level of Influence**	
Patient-Clinician Relationships	“I had a lot of problems getting in contact with that doctor (primary care doctor). And after, I think it was the first four months after the amputation, and I just kept on trying to contact her… and I would try to call her, and she never returned my calls.” (47-year-old Hispanic male, history of multiple DFUs, foot infection, and toe amputation)
	“They [the companies] make a big deal about bringing the nurse in and have them trained on me and then two weeks later, I get a new nurse and redo it.” (45-year-old White female, underwent more than 20 procedures for DFUs)
	“I see him once and a current situation came up, so I haven’t been able to see him since then. [Due to the pandemic] it has been phone interviews, so, I haven’t really developed any significant rapport with my current endocrinologist.” (41-year-old White male, history of recurrent DFUs and toe amputations).
**Community Level of Influence**	
Availability of Services	“The nurses themselves have been wonderful but their companies have been mainly touch-and-go with maintaining the supplies being delivered at an appropriate time” (41-year-old White male, Medicaid).
	The insoles that I went in for, that they prescribed for me, it took me a long time to get them. Probably like three months after … and then when I got them, they, they were very flimsy, they didn’t last. It took me awhile to get another pair, a better design of the ones that they had” (47-year-old Hispanic male, self-employed, commercial health insurance).
	“It was a difficulty because I am on the reservation and sometimes the medical things that I would need, like I said, insulin, the IV antibiotics, they wouldn't be able to come out here and do it. If I had lived in a city, then the people would come and get it done.” (38-year-old Native male, Medicare, rural Arizona).
**Societal Level of Influence**	
Quality of Care	“I don’t really remember (doctors) saying anything on ways to prevent other ulcers.” (38-year-old Native male, Medicaid and Indian Health Services).
	“Well, early on they didn’t look at my feet. Before I got the ulcer, they didn’t look at them. They would just instruct me to check my blood sugar. But then after the ulcer and when they cut off my toe, that’s when they started to check my feet.” (67-year-old Hispanic male, commercial insurance).
	“I went to the ER down here in XXX (a community hospital) and that was Friday (was discharged home) and then I saw my doctor on Monday and he sent me to XXX (a tertiary hospital) in Tucson.” (41-year-old White male, history of multiple DFUs and two toe amputations)
	‘I called my doctor…. She told me I want you to see an infectious disease doctor and have them put you on an IV antibiotic …. So, I get to the infectious disease doctor, and he says, ‘I’m not going to put you on antibiotic, it isn’t infected.’ So, that’s how I ended up with an amputation because he did not put me on any antibiotic. So, I went into the hospital, and they assigned me an infectious disease doctor and she came in, I’ll never forget this, and she started talking to me like I was stupid, and she goes, ‘You know you’re diabetic, you should’ve gone to a doctor right away …’ And I said, ‘… hold on a second here, I am a very intelligent person and yes, I did, I went to my own doctor who made an appointment for me to see an infectious disease doctor.” (71-year-old White female, history of multiple DFUs and toe amputations)

### Individual Level of Influence

#### Health literacy

While most participants were aware of the risks of foot infection and amputation, there were significant gaps in their health literacy that compromised their ability to make informed decisions about when and how to seek medical care. Most notably, although all participants had a history of DFUs, many were unfamiliar with the term “ulcer” and expressed confusion when interviewers asked questions using that term. This finding, which reflects poor communication by providers and medical staff, resulted in most participants using alternate terms such as “blister,” “callous,” “cut,” “infection,” and “injury” to describe their foot abnormalities. This confusion in terminology was critical, as many patients described not initially seeking medical care because they interpreted their foot abnormality to be a common, everyday problem rather than one warranting medical attention. As one participant described: “Nobody ever really said what I’m looking for just anything that is not normal, I guess. But like I said, I have never heard of a diabetic foot ulcer.” (57-year-old Hispanic male, history of DFU).

In addition, participants described gaps in their health literacy related to the specifics of foot ulcer progression and the appropriate management strategies to prevent amputation. Most participants did not have a solid understanding of warning signs for when medical care should be secured for foot problems or what type of medical care should be sought. One frustrated participant stated: “If I had gotten better, like a different type of information that they could’ve given me, that might’ve helped me improve this ulcer to be going away. From what I have been given, you know, it’s just hard. I don’t know if it’s my foot itself or if it’s the medication. I don’t know. I don’t know if I am a unique case, I know there are people out there that have one foot. And they are able to get, probably, their ulcer better” (29-year-old Native female, history of DFU and recurrent foot infection).

#### Insurance coverage

While all participants had medical care coverage under Medicaid, Medicare, Indian Health Services or commercial insurance, the majority described significant medical expenses and financial strain related to their diabetes care in general, and in many cases to DFU care in particular. Most of the participants reported multiple recurring expenses such as medications (particularly insulin), co-payments for specialist visits and procedures, and the need for extensive travel, a financial strain that was frequently exacerbated by temporary or permanent loss of employment and under-employment. One participant said that following his second toe amputation: “I was in the hospital for 15 days, 13 days. They are charging me a copay, but I don’t have money to pay it. I am currently not working. I have social security and they don’t give me very much and it’s not enough to cover the copay.” (67-year-old Hispanic male, commercial insurance). In addition, many described substantial out-of-pocket payments for ancillary supplies, such as diabetic footwear and wound dressings due to inadequate insurance coverage, which often resulted in participants being unable to secure the supplies and care they needed for optimal DFU management. For example, a participant explained: “They want me to get diabetic shoes and the orthotic but at the time I didn’t have Medicaid … and with the deductible, they wanted $1,000 for the pair of shoes and the orthotic and I couldn’t afford it.” (45-year-old White female, Medicaid).

### Interpersonal Level of Influence

#### Patient–Clinician Relationships

Participants reported a wide array of levels of satisfaction with their medical providers, from long-standing personal and medically supportive relationships to negative experiences of not being listened to or being bounced from provider to provider. A predominant theme involved fragmented relationships with healthcare providers due to multiple factors including patients’ changes in residence, transitions in insurance status, providers leaving the area or switching practices, providers’ medical and holiday leave, and the COVID-19 pandemic. Given the complexity of managing their diabetes and related complications, these interruptions to patient-clinician relationships posed considerable barriers to effective disease management.

In addition, participants mentioned challenges in making timely appointments, and in getting time with their primary care physicians after major clinical events such as hospitalizations. One patient explained: “I had a lot of problems getting in contact with that doctor (primary care doctor). And after, I think it was the first four months after the amputation, and I just kept on trying to contact her… and I would try to call her, and she never returned my calls.” (47-year-old Hispanic male, history of multiple DFUs, foot infection, and toe amputation).

Similar challenges existed around establishing trusting relationships with the nurses that conducted home wound care following DFUs and amputations. This was due in large part to turnover in nursing staff or the rotation of nurses who conducted their home visits. A participant explained: “They [the companies] make a big deal about bringing the nurse in and have them trained on me and then two weeks later, I get a new nurse and redo it.” (45-year-old White female, underwent more than 20 procedures for DFUs).

Lastly, participants reported that the COVID-19 pandemic further intensified this lack of provider continuity due to limited in-person visits. For example, one participant described his struggles to connect with a new endocrinologist during the pandemic, stating: “I see him once and a current situation came up, so I haven’t been able to see him since then. [Due to the pandemic] it has been phone interviews, so, I haven’t really developed any significant rapport with my current endocrinologist.” (41-year-old White male, history of recurrent DFUs and toe amputations).

### Community Level of Influence

#### Availability of Services

Participants commonly reported struggles with getting the medical equipment needed to prevent and manage their DFUs in a timely fashion, including offloading braces, dressing supplies, and therapeutic shoes and insoles. A few noted that the wound supplies provided by the hospital, clinic, or home healthcare companies ran out before their wounds had healed. One participant described maintaining medical supplies as his biggest challenge, saying: “The nurses themselves have been wonderful but their companies have been mainly touch-and-go with maintaining the supplies being delivered at an appropriate time” (41-year-old White male, Medicaid). Despite having prescriptions from physicians and insurance coverage, many participants also faced long waits for securing specialized diabetic shoes from medical supply companies, resulting in delayed or interrupted care. One participant described: "The insoles that I went in for, that they prescribed for me, it took me a long time to get them. Probably like three months after … and then when I got them, they, they were very flimsy, they didn’t last. It took me awhile to get another pair, a better design of the ones that they had” (47-year-old Hispanic male, self-employed, commercial health insurance).

Participants living in rural areas outside of Tucson cited additional challenges in managing their DFUs due to the time, expense, and distance involved in securing the elaborate routines of specialist appointments, routines, medications, and wound care necessary to effectively manage their DFUs. One participant described: “It was a difficulty because I am on the reservation and sometimes the medical things that I would need, like I said, insulin, the IV antibiotics, they wouldn't be able to come out here and do it. If I had lived in a city, then the people would come and get it done.” (38-year-old Native male, Medicare, rural Arizona).

### Societal Level of Influence

#### Quality of Care

Many participants noted insufficient preventative foot care and education prior to DFU onset. Some reported that they did not learn about ulcer prevention until they developed DFUs. For example, one participant stated: “I don’t really remember (doctors) saying anything on ways to prevent other ulcers.” (38-year-old Native male, Medicaid and Indian Health Services). Some participants similarly reported that they did not receive routine foot examinations prior to developing their first DFU, even though they had regularly scheduled primary care appointments. One explained: “Well, early on they didn’t look at my feet. Before I got the ulcer, they didn’t look at them. They would just instruct me to check my blood sugar. But then after the ulcer and when they cut off my toe, that’s when they started to check my feet.” (67-year-old Hispanic male, commercial insurance).

Other barriers presented themselves while seeking adequate medical care for their new ulcers. Participants initially sought care from a variety of different venues— primary care doctors, podiatrists, specialists, emergency rooms, and urgent care clinics— as determined by how serious they interpreted their foot problems and insurance status and access issues. Some participants had the experience of being sent to multiple facilities in search of appropriate care, and those living in rural areas faced travel to different cities or towns. For example, a participant recalled that: “I went to the ER down here in XXX (a community hospital) and that was Friday (was discharged home) and then I saw my doctor on Monday and he sent me to XXX (a tertiary hospital) in Tucson.” (41-year-old White male, history of multiple DFUs and two toe amputations).

Many respondents experienced initial misdiagnoses and delays in receiving care. This included a few participants who presented for diabetic foot complications to acute care facilities, such as urgent care clincs and emergency rooms, and were sent home without an appropriate diagnosis, treatment, and follow-up. One woman recalled her frustrating journey that led to amputation:

‘I called my doctor…. She told me I want you to see an infectious disease doctor and have them put you on an IV antibiotic …. So, I get to the infectious disease doctor, and he says, ‘I’m not going to put you on antibiotic, it isn’t infected.’ So, that’s how I ended up with an amputation because he did not put me on any antibiotic. So, I went into the hospital, and they assigned me an infectious disease doctor and she came in, I’ll never forget this, and she started talking to me like I was stupid, and she goes, ‘You know you’re diabetic, you should’ve gone to a doctor right away ...’ And I said, ‘… hold on a second here, I am a very intelligent person and yes, I did, I went to my own doctor who made an appointment for me to see an infectious disease doctor.” (71-year-old White female, history of multiple DFUs and toe amputations)

## Discussion

Over the past two decades, substantial advances in diabetes therapy have greatly extended health and reduced morbidity. However, as evidenced in this article, significant obstacles to effective DFU treatment and management remain at multiple levels of the healthcare system. Some of these obstacles can be mitigated with more thoughtful education and alignment of access points to receive adequate health care. In this context we offer observations from our study to help address these deficits, particularly as they relate to decreasing notable health disparities.

An important individual level barrier is deficits in health literacy surrounding appropriate terminology to describe diabetic foot complications and how to make informed medical decisions about when to seek medical intervention [25]. Our findings suggest that a more aggressive and tailored education approach that guides patients to act quickly in seeking medical care and for rapid wound examination is warranted. Part of this education needs to emphasize that diabetes increases the infection and amputation risks of these seemingly “minor” foot injuries. Burdensome expenses related to DFU care posed a second individual level barrier, suggesting the need for continued advocacy for full coverage of DFU care among safety net insurance providers [26, 27].

On the interpersonal level, our data illustrate that disruptions to the patient-clinician relationship damages rapport with patients and hinders optimal DFU care. Study participants frequently reported difficulties in accessing appropriate health care providers and disruptions to the patient-physician relationship due to the turnover of providers, changes to region and insurance status, and other factors. This gap calls for developing solutions to address medical provider shortages and to “fill in” health care assessment in a timely manner. One potential approach is to expand the use of trained community health workers who can help triage persons with differing levels of foot ulcers to available health care providers who work outside of the patient’s known environment [28, 29].

On the community level, despite having appropriate prescriptions and insurance coverage, participants described significant challenges receiving medical equipment, which was often perceived to be due to shortcomings at the medical supply companies. Since most persons with diabetes see their pharmacist more frequently than any other member of their health care team, developing collaborations between pharmacies, providers, or healthcare system in which pharmacists take on the role of providing medical equipment such as wound care supplies or diabetic shoes, may be an effective approach. Pharmacist supported diabetes care has been shown to be well received by minority patients and to result in improved diabetes outcomes [30, 31].

Finally, on the societal level, there is a need to improve preventive care for DFUs on the primary care physician level, a crucial strategy for limb salvage. The American Diabetes Association recommends that all patients with diabetes have their feet inspected at each doctor visit and have a comprehensive foot evaluation at least annually to identify risk factors for DFUs [32]. Greater focus needs to be placed on educating medical providers and patients, and on the importance of preventive foot care including self-foot inspection, foot examination by a medical professional, and the use of appropriate footwear. In addition, given that sample participants commonly reported receiving misdiagnoses and delays after seeking medical care for DFUs, a standardized protocol and care pathway for when, where, and how patients should seek initial DFU care and how the DFUs should be treated are imperative. Because delays occur both before and after seeking care, a focus must be made to educate both patients and providers about the standard protocol [33].

There are limitations to this study which should be considered when interpreting the results. Given the relatively modest sample size, we were not able to analyze the data for gender or age effects or by duration of diabetes. Nonetheless, this hard to reach patient sample representing a diverse population did offer very similar stories about the experiences and health disparities they faced in dealing with DFUs.

## Conclusions

Diabetic foot ulceration remains a common and life-altering disease complication and one that disproportionately burdens people of racial and ethnic minority status, low socio-economic status, low insurance coverage, and those residing in rural areas. Our study examined the lived experience of a sample of persons with diabetes that face significant barriers at all levels of the healthcare system. Their stories highlight the importance of selecting multiple points of entry to make significant improvements in peoples’ health literacy, relationships with providers, and access to quality and effective medical care, services, and medical supplies. Moreover, this approach should creatively incorporate multiple possible modes of service delivery, including the integration of community health workers and pharmacists. While there are considerable challenges to achieving this goal, concerted efforts are needed to reduce DFUs’ devastating effects on mortality and morbidity and the inequitable burden of poorly managed diabetes foot care among highly affected populations.

## Data Availability

The de-identified qualitative data that support the findings of this study are available from corresponding author upon reasonable request.
